# Secret Remedies

**Published:** 1885-03

**Authors:** 


### SECRET REMEDIES.

Secret Medical preparations may be good, but they may be injurious. The system should be discouraged. Particularly should mothers refuse to give their children remedies, the ingredients of which they and their physicians do not know. Many remedies for children are absolutely dangerous. Syrup of White Poppies Soothing Syrup and Parégoric all contain Morphine or Opium in some form. The vegetable Castoria however has acquired its popularity as a corrector of children's ailments, not alone because it is a reliable medicine, but from the fact that its label inspires confidence. We shall hail the time when Latin prescriptions and secret remedies cease to be the means through which unprincipaled Empirics may impose upon a credulous public.

